# Chemical Defence by Sterols in the Freshwater Ciliate *Stentor polymorphus*

**DOI:** 10.3390/biology11121749

**Published:** 2022-11-30

**Authors:** Federico Buonanno, Francesco Trenti, Gabriele Achille, Adriana Vallesi, Graziano Guella, Claudio Ortenzi

**Affiliations:** 1 Laboratory of Protistology and Biology Education, Department of Education, Cultural Heritage, Tourism (ECHT), University of Macerata, 62100 Macerata, Italy; 2 Bioorganic Chemistry Laboratory, Department of Physics, University of Trento, 38050 Trento, Italy; 3 Laboratory of Eukaryotic Microbiology and Animal Biology, School of Biosciences and Veterinary Medicine, University of Camerino, 62032 Camerino, Italy

**Keywords:** Stentor, predator-prey interactions, sterols, ergosterol, ergosterol peroxide

## Abstract

**Simple Summary:**

Predator-prey interactions are widely observed in nature and involve not only animals but also unicellular organisms. In this context, the basic techniques for offence or defence adopted by predators and prey can be mediated by mechanical/morphological and/or chemical strategies. Among unicellular organisms, ciliated protozoa represent a paradigmatic model for the investigation of both mechanisms. Therefore, in this study, we choose to investigate the defensive functions adopted by the ciliate *Stentor polymorphus* against predators that share the same aquatic ecosystem. On the basis of the experimental observations, we found that the defensive strategy evolved by this ciliate is essentially chemical and mediated by a mix of sterols. The defensive function of these molecules appears as a novelty, at least among the ciliated protozoa. Overall, our study represents an advance in the knowledge of the ethology and ecology of ciliates, with particular regard to the natural compounds that this group of organisms has selected in the last 1.8 billion years.

**Abstract:**

Heterotrich ciliates typically retain toxic substances in specialized ejectable organelles, called extrusomes, which are used in predator-prey interactions. In this study, we analysed the chemical defence strategy of the freshwater heterotrich ciliate *Stentor polymorphus* against the predatory ciliate *Coleps hirtus*, and the microturbellarian flatworm *Stenostomum sphagnetorum*. The results showed that *S. polymorphus* is able to defend itself against these two predators by deploying a mix of bioactive sterols contained in its extrusomes. Sterols were isolated in vivo and characterized by liquid chromatography-mass spectrometry (LC-MS), and nuclear magnetic resonance (NMR), as ergosterol, 7-dehydroporiferasterol, and their two peroxidized analogues. The assessment of the toxicity of ergosterol and ergosterol peroxide against various organisms, indicated that these sterols are essential for the effectiveness of the chemical defence in *S. polymorphus.*

## 1. Introduction

*Stentor polymorphus* is a large trumpet-shaped contractile (stretched individuals grow up to 2 mm long) colourless freshwater ciliate, belonging to the order Heterotrichida. Commonly present in ponds, wells, and lakes, this ciliate is also capable of attaching itself to aquatic objects by means of a specialized posterior holdfast organelle secreting a sticky substance [1].

As in the case of other heterotrich ciliates, the cortex of *S. polymorphus* is characterized by the presence of membrane-bound, colourless cortical granules (a kind of extrusomes), with a diameter of ~0.5–1 µm [1]. As is common in heterotrichs, extrusomes discharge their content outside of the cell as a reaction to external stimuli that can occur during the predator-prey interactions with other protists and/or microinvertebrates [2].

To date, colourless cortical granules have been found in heterotrics, such as *Blepharisma hyalinum*, *Climacostomum virens*, *Spirostomum ambiguum* and *Spirostomum teres*. For *B. hyalinum*, the content of the cortical granules has not been chemically characterized, and its protective function are only assumed. Conversely, the 5-alkylresorcinol climacostol 1,3-dihydroxy-5-[(*Z*)-non-2′-enyl]benzene, the mono-prenyl hydroquinone (2-(3-methylbut-2-enyl)benzene-1,4-diol), and the spirostomin (spiro[(2,5-dimethyl-5,6,7,8-tetrahydronaphthalene-1,4-dione)-8,6′-(pyrane2′,5′-dione)]), have been, respectively, isolated from the extrusome discharges of *C. virens*, *S. ambiguum*, and *S. teres*, and structurally characterized [3,4,5,6].

Climacostol, mono-prenyl hydroquinone and spirostomin have been reported as toxic compounds able to assure the protection against predators and, at least for climacostol, to assist/facilitate the prey ingestion [2].

With regard to the genus *Stentor*, the blue pigment stentorin, obtained from crude extracts of *S. coeruleus* cells, has been structurally characterized as 2,2′,4,4′,5,5′,7,7′-octahydroxy-3,3′-diisopropylnaphthodianthron, and chemically synthesized [7,8,9,10,11]. Miyake et al. demonstrated that stentorin is contained in extrusomes and can mediate the chemical defence against raptorial ciliates [11]. Later, they showed that colourless cortical granules of *S. polymorphus* are also extrusive organelles [3]. It was thus hypothesized that these granules contain toxic material which is able to repel and kill the predatory ciliate *Dilpetus margaritifer*, strongly suggesting the defensive function of these organelles [3]. It was observed that when *D. margaritifer* recognizes a cell of *S. polymorphus* as a prey and attacks it by discharging its toxicysts, *S. polymorphus* repels the predatory ciliate by discharging some toxic material from its extrusive cortical granules, which induces a rapid backward swimming in *D. margaritifer.* In addition, the toxic material collected from massive cultures of *S. polymorphus* exerts cytotoxic effects against the predator (unpublished data, see [3]).

In this study we demonstrate that the cortical granules of *S. polymorphus* are true extrusomes used for chemical defence. In addition, we isolate and characterize the toxic compounds contained in extrusomes, and evaluate the effectiveness of the purified molecules in the *S. polymorphus* defence strategy against predators.

## 2. Material and Methods

### 2.1. Organisms and Culture Methods

*Stentor polymorphus* stock GF-1 was collected in a pond in Cessapalombo MC, Italy (see Acknowledgments). Cells are maintained in the balanced salt solution (SMB) [4], or in Jaworski’s medium (JM) solution [12], and fed with the flagellate *Chlorogonium elongatum*. The species determination was based on both the morphological and molecular analyses. *S. polymorphus* was originally green, due to endosymbiotic algae, but white clones were obtained by culturing in the dark, in order to reduce the possible interference of the symbionts in the experiments.

*Blepharisma japonicum* strain R1072, *Coleps hirtus* clone PC-4, *Euplotes aediculatus*, *Paramecium multimicronucleatum* clone TL-2, *Paramecium tetraurelia* stock 51 were also cultured in SMB and fed with *C. elongatum*. *Spirostomum ambiguum* stock Pol-5 and *Spirostomum teres* stock Pol-1 were cultured in bacterized culture medium [13,14].

*Stenostomum sphagnetorum* (Platyhelminthes: Turbellaria), a common freshwater predator of ciliates, was cultured as described by in previous work [13]. 

### 2.2. Small Subunit (SSU) rRNA Gene Amplification and Sequencing

To obtain the full-length SSU rRNA gene sequence, the universal eukaryotic forward primer 5′-CTGGTTGATCCTGCCAG-3′ [15] and the hypotrich-specific 18S reverse primer 5′-TGATCCTTCYGCAGGTTC-3′ [16] were used in the PCR amplifications. Reactions were performed by adding cell lysates, obtained by boiling Five starved cells in 5 µL of distilled water, to 50 µL (final volume) of the reaction mixture containing 2 mM MgCl_2_, 250 mM of dNTP, one unit of Phusion High-Fidelity Taq DNA polymerase (Thermo-Fisher Scientific Inc., Waltham, MA, USA), and 0.2 mM of each primer. The PCR amplifications were run in an Eppendorf Ep-gradient Mastercycler (Eppendorf, Hamburg, Germany), following a standard program (30 s of denaturation at 98 °C, 30 s of annealing at 60 °C, 40 s of extension at 72 °C, for 35 cycles), with a final extension step of 5 min at 72 °C. Amplicons were purified using the NucleoSpin PCR clean-up kit (Macherey–Nagel, Düren, Germany) and sequenced in both directions. To minimize the amplification errors, the amplicons of three different reactions were sequenced. The SSU rRNA gene sequence is deposited at the GenBank database with accession number OP379682.

### 2.3. Preparation of the Extrusome-Deficient Cells and Extrusome Discharges

Extrusome-deficient cells of *S. polymorphus* were obtained according to a protocol, based on a cold-shock induction of extrusome discharge [17]. Briefly, massive cultures of ciliates (cultured as described in Section 2.1) were grown to reach the concentration of about 20,000 cells/mL, were mixed with ice-cooled SMB in a 1:5 ratio, at 0 °C for 10 s, and then centrifuged at about 27× *g* to separate the cells from the supernatant. Pellets containing cells were washed twice, resuspended for 2 h in SMB at 23 °C, and then used in the experiments. The extrusome-deficient cells were healthy as control cells [17].

Supernatants containing the extrusomes’ discharge were collected, and to increase solubility of the hydrophobic molecules, pure methanol was added to obtain a final 2% methanol solution. This solution was stirred, dried under vacuum and stored at −20 °C until use.

### 2.4. Predator-Prey Experimental Design

Samples of five or 10 *S. polymorphus* cells, either untreated or extrusome-deficient cells, were mixed with 200 cells of *C. hirtus* or two specimens of *S. sphagnetorum* in 500 μL of SMB. Each mixture was made in six replicates, for both the untreated and extrusome-deficient cells of *S. polymorphus*, and observed under a stereoscopic microscope after 5 and 24 h, for the mixtures involving *C. hirtus*, and after 1 and 5 h, for the mixtures involving *S. sphagnetorum*. The data were reported as the means ± standard error (SE) of six independent determinations, and the significance of the differences between the mean values was examined by Student’s *t*-test with the significance threshold set at *p* < 0.05.

The time for the observations and the proportion between the numbers of the predatory organisms and prey were established in a set of preliminary mixtures to improve the efficient encounters for each used predator.

### 2.5. Toxicity Tests

Ergosterol (98%, Acros Organics, Shanghai, China) and ergosterol peroxide (98%, Cayman Chemical Company, Ann Arbor, MI, USA) were diluted in pure ethanol by heating the solutions at 60 °C with a slight stirring, to obtain 1 mM stock solution for each compound. Both solutions were stored at −20 °C and placed at room temperature for 1 h before being used in the experiments.

To evaluate the toxicity of ergosterol and its peroxide, triplicate samples of 10 ciliate cells or metazoan specimens were placed in depression slides containing 250 μL SMB and increasing concentrations of each sterol (from 1.5 to 200 μM). The number of surviving organisms (estimated on the basis of morphology and locomotion) was counted after 1 or 24 h. Median lethal concentrations (LC_50_) were then assessed on a concentration–survival curve [13], and extrapolated by a nonlinear regression analysis using GraphPad Prism 6 software (GraphPad Software, San Diego, CA, USA) with the confidence index of 95%.

### 2.6. Organic Extraction of Cell Pellets and the Extrusomes Discharge

Samples of *S. polymorphus* whole cell pellets, ciliate extrusomes, and the algal food *Chlorogonium* were used to prepare the organic extracts. The extraction was performed by adding 5 mL Chloroform:Methanol = 2:1 and sonicating thoroughly for 15 min. Raw extracts were cleaned from chlorophylls by C18 Solid Phase Extraction (SPE) column (Supelco; Supelclean ENVI-18 SPE tube, 3 mL), using 100% acetonitrile as an eluent. The compounds of interest were recovered by eluting with Methanol:Dichloromethane = 9:1.

### 2.7. LC-Electrospray Ionization-Mass Spectrometry Analysis and the Sterols Purification

*Stentor* total cells, extrusomes discharge, and algal extracts were analysed by liquid chromatography-mass spectrometry (LC-MS) (Model 1100 series; Hewlett-Packard, Palo Alto, CA, USA), coupled to a quadrupole ion-trap mass spectrometer (Esquire LCTM; Bruker, Bremen, Germany), equipped with an electrospray ionization source in both positive and negative ion modes. The chromatographic separation of sterols was carried out at 303 K on a C18 column (Kinetex 5 µ; length, 100 mm; particle size, 2.6 µm; internal diameter, 2.1 mm; pore size, 100 Å; Phenomenex, Torrence, CA, USA). The solvent system consisted of eluent A as MeOH/H_2_O (7:3, *v*/*v*), containing 10 mM ammonium acetate and eluent B as isopropanol/MeOH (10:90, *v*/*v*), containing 10 mM ammonium acetate. Samples were resuspended in 1 mL CHCl_3_/MeOH (2:1, *v*/*v*), and 10 µL was run with an isocratic gradient A:B = 15:85. The MS scan range was 13,000 U/s in the range of 50 to 1500 *m*/*z*, with a mass accuracy of ~100 ppm. The diode array detector was set at wavelengths 204; 282; and 665 nm. The nebuliser gas was high purity nitrogen at a pressure of 20 to 30 psi, at a flow rate of 6 L/min and at 300 °C. The electrospray ionization was operated in the positive ion mode. Data were processed using the DataAnalysis 3.0 software (Bruker Daltonik, Bremen, Germany). Sterols and peroxy-sterols were isolated by HPLC (Agilent, model 1100 series; Hewlett-Packard) using the isocratic gradient of 100% acetonitrile on a C18 column (RP18 Kinetex XB-C18, 4.6 × 250 mm; Phenomenex, Torrence, CA, USA) at a flow of 1 mL/min. The UV chromatograms were acquired at 280 nm.

### 2.8. Nuclear Magnetic Resonance Analysis

^1^H-NMR (400 MHz) spectra of sterols extract dissolved in CDCl_3_ were recorded at 300 K on a nuclear magnetic resonance (NMR) spectrometer (400 MHz; Bruker-Avance, Bremen, Germany), with a 5-mm broadband inverse probe (BBI) with pulsed-gradient field utility. The ^1^H-90° proton pulse length was 9.3 µs, with a transmission power of 0 db. The probe temperature was maintained at 300.0 K (±0.1 K) using a variable temperature unit (B-VT 1000; Bruker, Billerica, MA, USA). The calibration of the chemical shift scale (δ) was performed on the residual proton signal of the CDCl_3_ at δH 7.260 ppm. The following measurements were performed: ^1^H-NMR (i.e., proton chemical shifts, scalar couplings). The resulting 1D-NMR spectra were analysed using TopSpin 3.6.1 (Bruker, Bremen, Germany) and MestReNova v14.1 (Mestrelab Research, Santiago de Compostela, Spain) [18].

## 3. Results

### 3.1. Species Identification

The cells of stock GF-1 were assigned to the species *S. polymorphus* on the basis of both morphological and genetic data. The phenotypic traits analysed were the cell morphology, the macronucleus shape, extrusome type and presence/absence of green symbiontic algae. The SSU rRNA gene sequence of strain GF-1 (accession number OP379682) is 1637-bp long, with a G + C content of 45.1 analyzed on BLASTn, the GF-1 sequence shows the closest relatedness (100% identity, 100% query cover) to the SSU rRNA gene sequence (accession number AF357144.1) from a *S. polymorphus* strain isolated from China, and to the SSU rRNA gene sequence (accession number FN659823.1) from a *S*. *polymorphus* strain collected in Germany (99.9% identity, only one nucleotide substitution).

### 3.2. Interaction between the Stentor polymorphus and Coleps hirtus

To examine the behaviour of *S. polymorphus* upon exposure to the predatory ciliate *C. hirtus*, 10 cells of *S. polymorphus* and 200 cells of *C. hirtus* were mixed in 500 µL of SMB, and the interaction between the two organisms was observed by a stereomicroscope. During the first 10–15 min after mixing, the contacts between the predators and prey did not yield any particular effect, probably because *C. hirtus* was not able to promptly recognize the *S. polymorphus* as prey, as already observed for the interaction with *D. margaritifer* (Unpublished data, see [3]).

**Figure 1:**
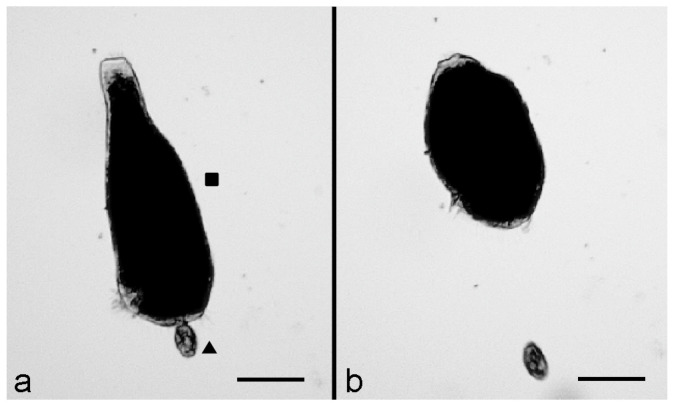
Predator-prey interaction between *C. hirtus* (▲) and *S. polymorphus* (■). (**a**) A cell of *C. hirtus* has an effective contact with a cell of *S. polymorphus* with its anterior portion. (**b**) The prey shows a contraction and becomes ovoid while the predator swims backwards. Photomicrographs obtained from a film clip. Scale bar = 100 µm.

Then, after 15–20 min, the interactions between the two organisms were effective, as indicated by the reaction of *S. polymorphus* which, immediately after contact with the predator, showed a rapid cell contraction and became ovoid (Figure 1a). This reaction was then followed by a fast backward swimming of the predator, which moved away from its prey (Figure 1b), suggesting the activation of a defensive mechanism by *S. polymorphus.*

**Figure 2:**
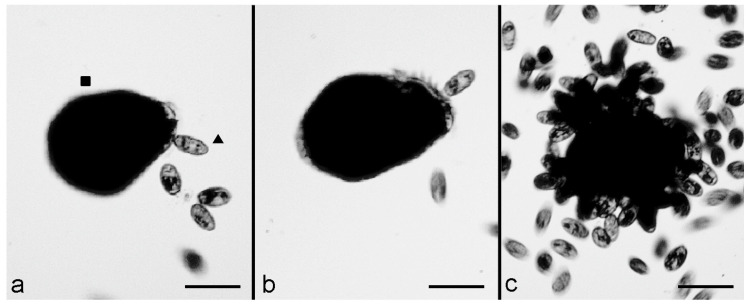
The predatory behaviour of *C. hirtus* (▲) on an extrusome-deficient cell of *S. polymorphus* (■). (**a**) A cell of *C. hirtus* has an effective contact with an extrusome-deficient cell of *S. polymorphus* with its anterior portion, and the prey immediately contracts. (**b**) The predator remains attached to the prey while other cells of *C. hirtus* contact the same prey. (**c**) the extrusome-deficient cell of *S. polymorphus* is fragmented and eaten by the predators. Photomicrographs obtained from a film clip. Scale bar = 100 µm.

To investigate the possible participation of the *S. polymorphus* cortical granules in this kind of interaction, the experiment was repeated by mixing 10 extrusome-deficient cells of *S. polymorphus* with 200 cells of *C. hirtus* in 500 μL of SMB. Furthermore, in this case, after 10–15 min, several effective contacts were observed, with the prey showing an immediate contraction after contact with the predator. However, the backward swimming of the predator was almost never observed, and *C. hirtus* remained attached to the prey (Figure 2a,b). Then, many *C. hirtus* cells attacked the same contracted prey, which was consequently fragmented and eaten by the group of predators (Figure 2c).

**Figure 3:**
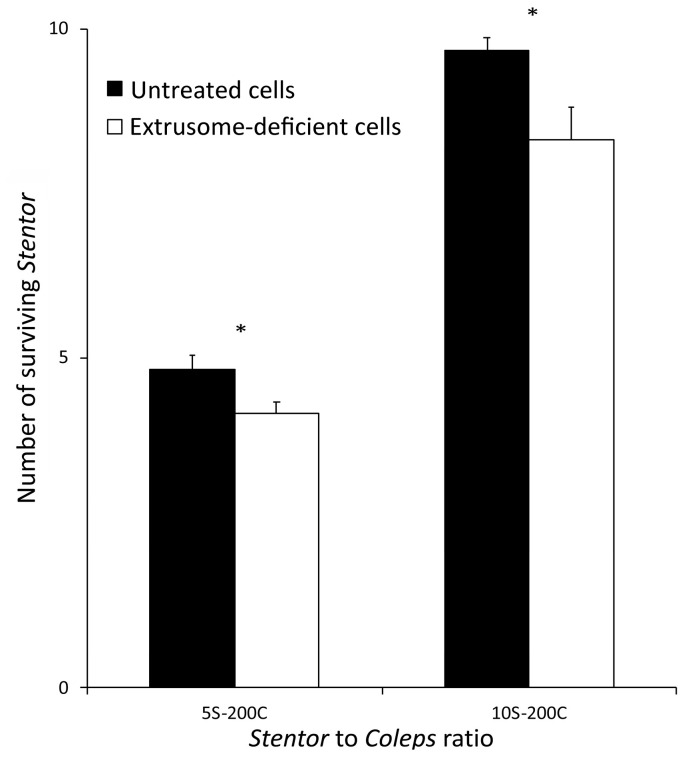
Effect of the extrusome deprivation in *S. polymorphus* on the predator-prey interaction with *C. hirtus.* Groups of five or 10 cells of either extrusome-deficient or untreated cells of *S. polymorphus* were mixed with groups of 200 cells of *C. hirtus* (the 5S-200C and 10S-200C, respectively). Each bar represents the mean (±SE) of six independent experiments. * *p* < 0.05.

To quantify the above observations, the parallel experiments were carried out with defined numbers of predators and prey, using both untreated and extrusome-deficient *S. polymorphus* cells and counting the number of *S. polymorphus* cells at 5 and 24 h after mixing with the predators. As reported in Figure 3*,* in all of the mixtures, the number of the untreated cells of *S. polymorphus* was significantly greater than the number of extrusome-deficient cells, thus implying that the defensive mechanism of *S. polymorphus* is essentially due to the discharge of the toxic material from extrusomes.

### 3.3. Interaction between the Stentor polymorphus and Stenostomum sphagnetorum

**Figure 4:**
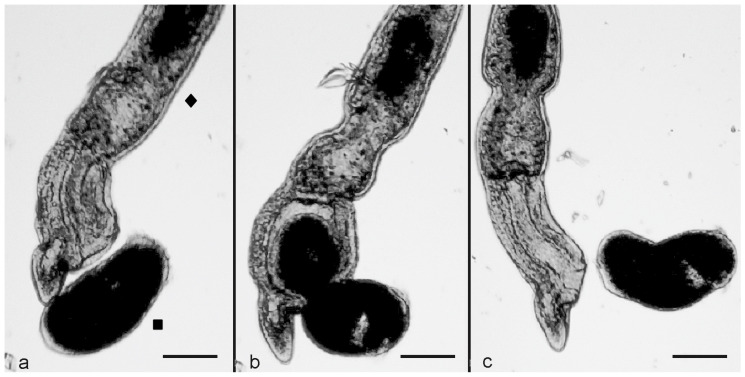
The predator-prey interaction between the microturbellarian *S. sphagnetorum* (◆) and *S. polymorphus* (■). (**a**) A specimen of *S. sphagnetorum* contacts a cell of *S. polymorphus* with its oral apparatus while the prey shows a quick contraction. (**b**) The predator partially engulfs its prey. (**c**) the contracted *S. polymorphus* cell is disgorged alive within one second. Photomicrographs obtained from a film clip. Scale bar = 100 µm.

To observe the interaction between the microturbellarian flatworm *S. sphagnetorum* and *S. polymorphus*, mixtures of 10 specimens of *S. sphagnetorum* and 100 cells of *S. polymorphus* were made in 500 μL of SMB and observed under a stereomicroscope. The effective interactions occurred when the frontal part of the flatworm came into contact with a *S. polymorphus* cell (Figure 4a). Then, the prey was partially swallowed by the predatory flatworm (Figure 4b) but it was disgorged almost instantly after ingestion, probably due to the discharge of content from the *S. polymorphus* extrusomes into the pharynx of *S. sphagnetorum* (Figure 4c).

The disgorged *S. polymorphus* cell swam away, apparently healthy or scarcely damaged. In rare cases, the *S. polymorphus* cell was broken, with a portion of the cell body retained inside the predator and the other disgorged. In such events, the ejected part of the prey was visibly damaged. These observations suggest that *S. polymorphus* utilizes a defensive mechanism against the turbellarian worm.

**Figure 5:**
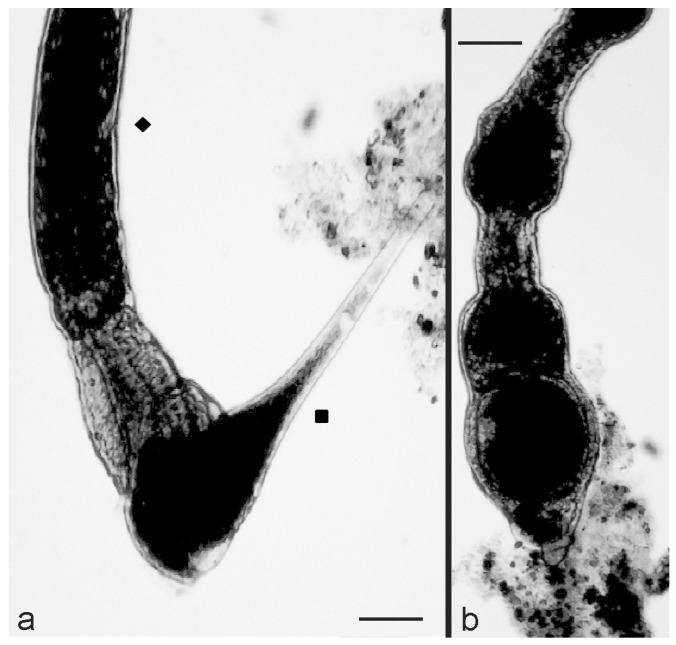
The predator-prey interaction between *S. sphagnetorum* (◆) and the extrusome-deficient cells of *S. polymorphus* (■). (**a**) the predator contacts an extrusome-deficient cell of *S. polymorphus.* (**b**) after a quick contraction, the prey is completely engulfed by the predator. Photomicrographs obtained from a film clip. Scale bar = 100 µm.

To verify the involvement of the extrusome discharge in the defensive mechanism of *S. polymorphus*, similar mixtures between predators and preys were prepared, but using extrusome-deficient cells as prey. It was observed that when a flatworm contacted an extrusome-deficient cell, the prey was usually taken into the pharynx of the predator and almost always fully ingested by the microturbellarian worm (Figure 5a,b).

**Figure 6:**
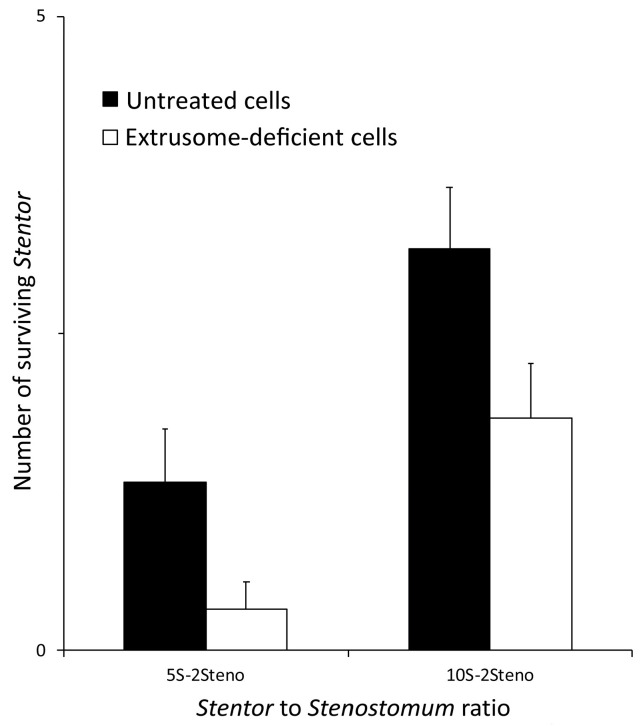
Effect of the extrusome deprivation in *S. polymorphus* obtained after the cold-shock treatment on the predator-prey interaction with *S. sphagnetorum*. Groups of five or 10 cells of either the extrusome-deficient (ED) or untreated cells of *S. polymorphus* were mixed with two specimens of *S. sphagnetorum* (the 5S-2Steno and 10S-2Steno respectively). Each bar represents the mean (±SE) of six independent experiments.

In the quantitative experiments performed by mixing a defined number of both *S. sphagnetorum* and *S. polymorphus* (two predators and five or 10 preys), only some untreated cells were consumed by the predatory flatworm, whilst a significant number of extrusome-deficient cells disappeared at 1 and 5 h after mixing (Figure 6).

These results support the assumption that the defensive mechanism of *S. polymorphus* against *S. sphagnetorum* is related to the discharge of toxic substances contained in the extrusomes.

**Figure 7:**
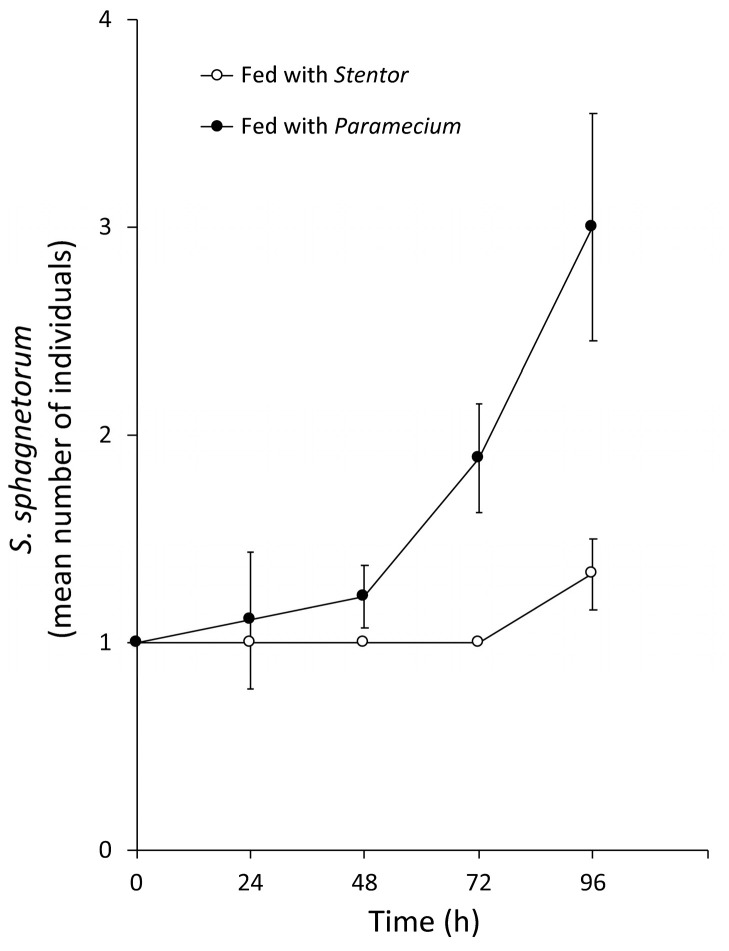
Comparison between the growth curves of *S. sphagnetorum* fed with *S. polymorphus* and fed with *Paramecium multimicronucleatum*. One *S. sphagnetorum* was mixed with 50 cells of *S. polymorphus* or with 100 cells of *P. multimicronucleatum*, a non-toxin producing ciliate as a control. The specimens of *S. sphagnetorum* were counted for 96 h. Each point denotes the mean (±SE) of nine independent tests.

To ascertain whether the predator is damaged by the toxins ingested with the prey, single specimens of *S. sphagnetorum* were fed with untreated *S. polymorphus* cells or with cells of *Paramecium multimicronucleatum* (a non-toxin producing prey used as a control) in 500 μL of SMB, and the number of *S. sphagnetorum* in each mixture was then counted at increasing time, for 96 h. As shown in Figure 7, the flatworms fed *S. polymorphus* were unable to grow and reproduce as specimens fed *P. multimicronucleatum*, supporting the fact that the chemical defence of *S. polymorphus* is very effective against *S. sphagnetorum.*

### 3.4. Characterization of the Extrusome Discharge of Stentor polymorphus

**Figure 8:**
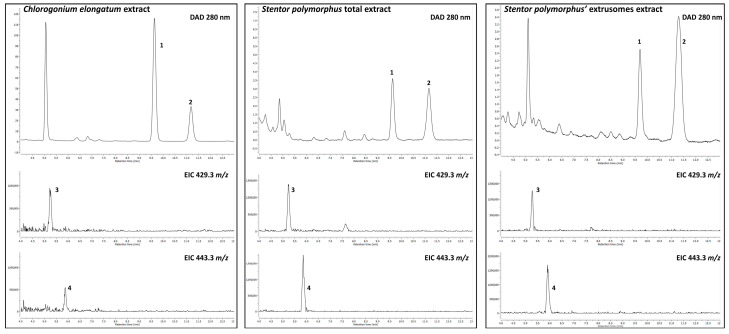
LC-UV-MS chromatograms of *Chlorogonium* (left panel), *S. polymorphus* cells (central panel) and the *S. polymorphus* extrusomes discharge (right panel) extracts. UV chromatograms are displayed for ergosterol **1** and 7-dehydroporiferasterol **2** (280 nm), while EIC traces are displayed for the oxidized analogues **3** and **4** (429.3 and 443.3 *m*/*z*, respectively).

**Figure 9:**
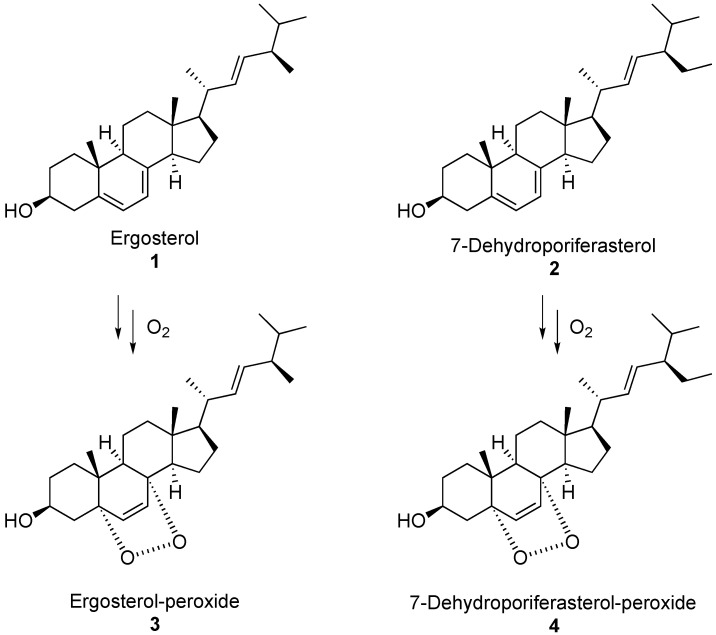
Structures of the sterols and their cyclic peroxides.

The organic extracts of *S. polymorphus*, the *S. polymorphus* extrusomes, and the green algae *Chlorogonium elongatum*, were first analysed by LC-MS, then the compounds of interest were isolated and analysed by ^1^H-NMR. All samples presented ergosterol **1**, 7-dehydroporiferasterol **2** and a minor amount of their corresponding cyclic peroxides (Figure 8 and Figure 9). The sterol peroxides were detected by extracted ion chromatograms (EICs) at *m/z* 429.3 for ergosterol peroxide **3** and at *m/z* 443.3 for 7-dehydroporiferasterol peroxide **4**.

**Figure 10:**
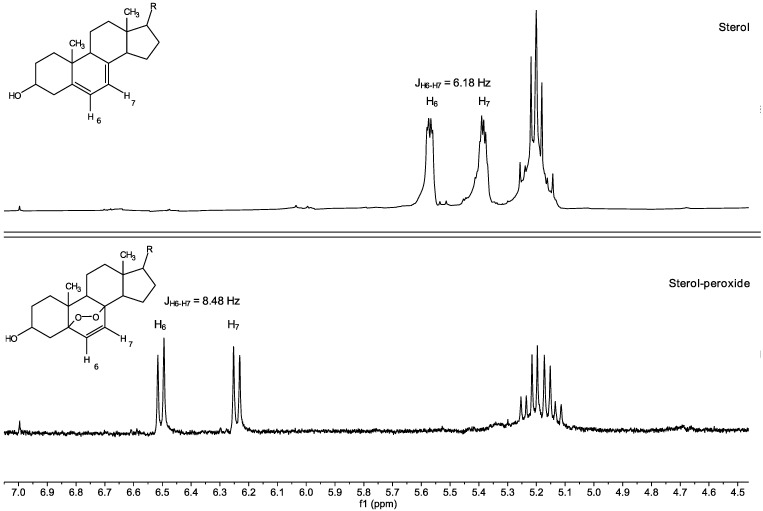
Key ^1^H-NMR signals of protons H6 and H7 in ergosterol (top panel) and its peroxidized analogue (bottom panel).

Although the purification of compounds **1** and **2** was challenging, we were able to univocally identify the major compound **1** as ergosterol by comparing the retention time, UV, and ^1^H-NMR spectra to the commercially available standard. Compound **2**, eluting in a minor chromatographic peak, was found to have superimposable UV and ^1^H-NMR spectra to ergosterol **1**, but an MS analysis suggested the presence of an extra methylene group (-CH_2_-) on the side chain. Based on the literature, this C29 sterol was assumed to be 7-dehydroporiferasterol **2** [19,20]. Two compounds **3** and **4** were detected by MS at *m/z* of +32 Da higher than ergosterol **1** and 7-dehydroporiferasterol **2**, suggesting the presence of an *endo*-peroxide cycle on ring A of the two sterols. The peroxide structures of these compounds were further confirmed by the ^1^H-NMR analysis (Figure 10), which showed the characteristic AB spin system of protons H6 and H7 [21].

Peroxidation occurs spontaneously, as we observed the purified ergosterol **1** and 7-dehydroporiferasterol **2** turned into their oxidized analogues in a few hours [22]. Therefore, it is difficult to establish whether the oxidized compounds are true metabolites of *S. polymorphus*, or artifacts. However, as reported by Andrade and Luo [23,24], and based on the biological tests performed in this study on the cytotoxicity of ergosterol **1** and ergosterol-peroxide **3**, these cyclic peroxides can be considered effective components of the extrusome content.

### 3.5. Toxicity of Ergosterol and Ergosterol Peroxide on the Ciliates and Stenostomum sphagnetorum

The toxic effect of *Stentor* sterols was evaluated on a panel of ciliates (one predator, six preys or competitors of *S. polymorphus*) and on the microturbellarian predator *S. sphagnetorum*, all sharing the same freshwater environment as *S. polymorphus*. Due to the extreme difficulty in isolating and purifying 7-dehydroporiferasterol **2** and its oxidized form in appreciable amounts, and to the commercial unavailability of these sterols, only ergosterol **1** and ergosterol-peroxide **3** were used in the assays.

Values of toxicity were estimated for each species exposed to increasing concentrations of sterols for 1 h and 24 h (Table 1). The two compounds were toxic for all of the tested species, with the cyclic peroxide proving more effective than ergosterol **1** after both 1 and 24 h of incubation.

The turbellarian flatworm *S. sphagnetorum*, the only multicellular organism used in the assay, showed the highest resistance to both sterols. Among the ciliates, *S. ambiguum* was found to be particularly resistant (LC_50_ values of 16.40 µM and 36.97 µM for, respectively, ergosterol-peroxide **3** and ergosterol **1**). As expected, *S. polymorphus* is also very resistant to its own toxins (LC_50_ values ranging between 31.7 µM and 38.9 µM for the two compounds). In contrast, the species which showed a particular sensitivity to the toxins are *C. hirtus*, *P. tetraurelia*, and *S. teres*, with LC_50_ values spanning from 5.37 µM (ergosterol-peroxide **3**) and 13.42 µM (ergosterol **1**).

## 4. Discussion

In this study, we report the isolation, purification, and structural characterization of four sterols contained in cortical granules of the ciliated protozoan *S. polymorphus* (Alveolata): ergosterol, ergosterol-peroxide, 7-dehydroporiferasterol, and 7-dehydroporiferasterol-peroxide. In addition to the most common functions reported for sterols (control of the membrane fluidity and permeability, the precursors of hormones, steroids and fat-soluble vitamins) [25,26], we demonstrate that at least ergosterol and its cyclic peroxide are used by the ciliate in its defence strategy against predators.

### 4.1. Chemical Defence in Stentor polymorphus

The results of the predator-prey interactions between *Stentor polymorphus* and the unicellular or multicellular predators reported in this study clearly reveal that the effectiveness of the defensive mechanism observed in *S. polymorphus*, depends on the release of toxic material from its extrusomes. In fact, only cells normally equipped with their extrusomes can successfully defend themselves from the raptorial ciliate *C. hirtus* or the carnivorous flatworm *S. sphagnetorum*, whereas the extrusome-deficient cells are easily attacked and eaten by the two predators.

In addition, the chemical defence practiced by *Stentor* is not only functional in deterring or preventing predator attacks, but also to develop a post-ingestion toxicity. This is particularly evident when the exponential growth curve of *S. sphagnetorum* fed with Paramecium is compared with the almost flat growth curve of the turbellarian fed with *Stentor*. *S. sphagnetorum* remains substantially incapable of reproducing for at least three days when it ingests one or more intact *Stentor* cells, similarly to what was observed in the predator-prey interactions between *S. sphagnetorum* and the toxic ciliate Pseudokeronopsis erythrina [27]. It is worth noting that, when the predator-prey interactions involve the same flatworm more than once, the predator blocks the attacks, ignoring the subsequent encounters with *S. polymorphus*. This characteristic behaviour of the prey selection performed by *S. sphagnetorum* has been already observed against other toxic prey [2], and suggests a further defensive mechanism adopted by *S. polymorphus* to increase its chance of surviving and escaping ingestion.

### 4.2. Defensive Sterols of the Stentor polymorphus

Spectroscopic measurements of compounds contained in the *S. polymorphus* extrusomes revealed the presence of ergosterol and 7-dehydroporiferasterol along with their oxidized analogues. Extracts of the green alga *C. elongatum* used as food have the same sterol composition, suggesting that all four compounds present in the extrusomes may derive from the *S. polymorphus* diet, rather than from the ciliate endometabolism (Figure 8). However, the molar ratio of ergosterol and 7-dehydroporiferasterol in the green alga (3:1 molar ratio) is remarkably different from that measured in the *Stentor* cells and extrusome-discharge (1:1 molar ratio), suggesting a tuned storage of these compounds in *S. polymorphus* membranes and extrusomes.

A similar trend was observed for the oxidized analogues ergosterol-peroxide and 7-dehydroporiferasterol-peroxide, that were present in 1:1 molar ratio in *Stentor* cells and extrusomes and 2:1 in the alga (Figure 8). The molar ratio was estimated by calculating the proportion between the UV peak integrals for sterols (ergosterol:7-dehydroporiferasterol) and the proportion of EIC peak area for sterols-peroxides (ergosterol-peroxide:7-dehydroporiferasterol-peroxide) (Figure 9). Unfortunately, we could not determine the molar ratio between sterols and their cyclic peroxides as their response factors for UV and MS ionization are not comparable. From these observations, it is possible to hypothesize that *S. polymorphus* incorporates sterols and peroxy-sterols from its diet and uses them as a chemical weapon against its prey and predators by exploiting their cytotoxic proprieties [28,29]. A similar situation has been described for two other species of algivorous ciliates, *Balanium planctonicum* and *Urotricha farcta*, for which the sterol composition did not resemble that of *Cryptomonas phaseulus*, their algal food. Ergosterol was the predominant sterol in *Cryptomonas*, whereas the stigmasterol was dominant in both ciliates. The authors supposed that the differences in the sterol composition between ciliates and their algal food may be due to species-specific sterol metabolism, accumulation, assimilation, or synthesis [30]. Nevertheless, the inability of the ciliates to synthesize sterols was stated by Desmond and Gribaldo [31], who carried out an extensive analysis of the taxonomic distribution and phylogeny of the enzymes of the sterol pathway in eukaryotic lineages.

### 4.3. Toxicity of the Stentor Sterols: Ergosterol and Ergosterol Peroxide

Dose-response experiments have been performed to evaluate the toxicity of ergosterol and ergosterol-peroxide on various ciliates and one metazoan, that can share the same microhabitat with *S. polymorphus*. Overall, the effects of ergosterol and ergosterol-peroxide on ciliates and the turbellarian flatworm confirm the results obtained in the predator-prey experiments. As expected, *S. polymorphus* shows the lowest sensitivity to its sterols, with no appreciable difference between the LC_50_ values of ergosterol and ergosterol-peroxide. On the contrary, as previously reported for the mono-prenyl hydroquinone (the toxin of *S. ambiguum*), the lowest values of LC_50_ were measured for *C. hirtus* and *P. tetraurelia*, two non-toxic species that adopt the mechanical defence mediated by calcareous armour or trichocysts, respectively [2].

Similarly to what has been observed for other toxins, such as climacostol and erytholactones [27], the concentrations of both sterols used to reach the LC_50_ values in *S. sphagnetorum* are the highest. This is not surprising, considering that for the multicellular organisms, long exposure times and/or high concentrations are necessary before tissues can be reached and injured by ciliate toxins, as also reported for the rotifers and ostracods [2]. In particular, *S. sphagnetorum* is furnished with secretory glands (the rhabdoids) located in the epithelium, which exert a defensive role in repelling the predators by discharging a mucous material [2]. Furthermore, at least in one case, it was also demonstrated that the mucus is used to neutralize the toxic substances used by predators, such as the raptorial ciliate, *D. margaritifer* [2].

### 4.4. Activity of Ergosterol and Ergosterol Peroxide on Parasitic Protozoa and Other Pathogens

To the best of our knowledge, our study is the first to report on the effects of ergosterol and ergosterol-peroxide on free-living protozoa and microinvertebrates, whereas the activity of both sterols against parasitic protozoa, pathogenic bacteria and viruses is well documented.

The permeabilization of the plasma membrane, the generation of ROS, depolarization of mitochondrial membrane potential, and parasite death were observed in *Trypanosoma cruzi* after treatment with ergosterol [32] or ergosterol-peroxide [33] at micromolar concentrations comparable with those used in this study. Additionally, ergosterol-peroxide proved to be the more active sterol against *Trypanosoma*, with a value of IC_50_ of 6.7 µg/mL (15.6 µM), within the range of LC_50_ reported in this study against free-living ciliates.

The effect of ergosterol-peroxide on *Entamoeba histolytica* was also investigated and resulted in a strong antiparasitic activity (IC_50_ = 4.23 nM), possibly due to the oxidizing effect on the parasitic membrane [34].

The antibacterial activity of ergosterol against *Enterococcus faecalis* and *Staphylococcus aureus* was demonstrated by Tran [35], with values of MIC of 16 µg/mL and 64 µg/mL, respectively. Ergosterol-peroxide was also evaluated for its antibacterial activity against *Mycobacterium tuberculosis*, on which it showed a significant effect of growth inhibition [36].

With regard to the antiviral activity, ergosterol-peroxide inhibits the progression of the porcine deltacoronavirus infection by suppressing the virus-induced autophagy in LLC-PK1 cells, the key process that facilitates virus replication [37].

## 5. Conclusions

To sum up, the data collected in this study indicate that *S. polymorphus* adopts a chemical defence against single-celled and/or multicellular predators, that is mediated by a mix of four sterols contained in its membrane-bound extrusomes. Two of the four sterols isolated and characterized, ergosterol **1** and its peroxide **3**, demonstrated to exert a cytotoxic effect against a set of free-living protozoa comprising the raptorial ciliate *C. hirtus*, and against the microturbellarian *S. sphagnetorum*, organisms that share the same microhabitat as *S. polymorphus*. Unfortunately, we did not collect any data about the other two sterols, 7-dehydroporiferasterol **2** and its oxidized form **4**, but considering that also these compounds are stored inside the extrusomes, we can speculate that they also may contribute to the chemical defence of the title ciliate.

## Figures and Tables

**Table 1:** Comparison of the cytotoxic effects of ergosterol and ergosterol peroxide on seven ciliate species and the microturbellarian *S. sphagnetorum*. Viability was assessed after 1 or 24 h of incubation and the LC_50_ values were obtained by a nonlinear regression analysis of three independent experiments with the 95% confidence limits calculated using GraphPad Prism 6 software.

Toxicity (LC_50_ μM; 95% C.I.)				
	Ergosterol		Ergosterol Peroxide	
**Ciliated protists**	1 h	24 h	1 h	24 h
*B. japonicum*	**16.80** (14.77–19.12)	**14.16** (13.52–14.83)	**13.34** (10.88–16.35)	**10.91** (9.74–12.22)
*C. hirtus*	**13.42** (1.12–16.2)	**9.87** (5.57–17.51)	**11.04** (7.91–15.39)	**6.51** (5.97–7.11)
*E. aediculatus*	**30.97** (23.71–40.45)	**25.33** (24.83–25.84)	**24.96** (22.58–27.60)	**8.78** (3.94–19.58)
*P. tetraurelia*	**11.24** (5.57–22.71)	**8.63** (6.75–11.04)	**6.26** (5.81–6.75)	**5.37** (5.36–5.38)
*S. ambiguum*	**36.97** (32.05–42.66)	**23.13** (13.68–39.11)	**27.64** (20.25–37.73)	**16.40** (10.14–26.52)
*S. teres*	**11.52** (11.14–11.91)	**11.31** (8.09–16.79)	**11.06** (9.00–13.60)	**9.47** (3.70–24.19)
*S. polymorphus*	**38.90** (38.48–39.33)	**33.56** (33.42–33.71)	**37.33** (37.28–37.33)	**31.70** (31.62–31.79)
***S. sphagnetorum***	**97.07** (96.35–97.79)	**74.48** (63.45–87.43)	**94.81** (94.75–94.86)	**44.34** (40.47–48.57)

## Data Availability

The data presented in this study are available upon reasonable request from the corresponding authors. The data are not publicly available due to privacy.
